# Risk of Myocardial Infarction in Patients With Aortic Stenosis: Insights From the VALVENOR Registry

**DOI:** 10.1016/j.jacadv.2025.101707

**Published:** 2025-04-25

**Authors:** Gilles Lemesle, Augustin Coisne, Sandro Ninni, Samy Aghezzaf, Basile Verdier, Guillaume Schurtz, Arnaud Sudre, Thomas Modine, Amine Tazibet, Bart Staels, David Montaigne, Christophe Bauters, VALVENOR investigators

**Affiliations:** a Heart and Lung Institute, University Hospital of Lille, CHU Lille, Lille, France; b University of Lille, Lille, France; c Institut Pasteur of Lille, Inserm U1011-EGID, Lille, France; d FACT (French Alliance for Cardiovascular Trials), Paris, France; e University of Lille, Inserm, CHU Lille, Institut Pasteur de Lille, Lille, France; f Clinical Trials Center, Cardiovascular Research Foundation, New York, New York, USA; g University of Lille, Inserm, CHU Lille, Institut Pasteur de Lille, Lille, France; h Heart and Lung Institute, University Hospital of Lille, CHU Lille, Lille, France; i Department of Cardiac Surgery, CHU Bordeaux, Hôpital Cardiologique Haut Leveque, Pessac, France; j University of Lille, Inserm, CHU Lille, Institut Pasteur de Lille, Lille, France

**Keywords:** aortic stenosis, coronary artery disease, myocardial infarction

## Abstract

**Background:**

A close interaction between aortic stenosis (AS) and coronary artery disease has been suggested. However, the risk of myocardial infarction (MI) in patients with AS is poorly described outside the context of aortic valve replacement.

**Objectives:**

The purpose of this study was to assess the incidence, correlates, and impact on outcomes of MI occurrence in patients with different degrees of AS severity.

**Methods:**

Between 2016 and 2017, the multicenter prospective VALVENOR registry enrolled 2,830 outpatients with native valvular AS (peak aortic jet velocity [Vmax] ≥2.5 m/s). AS was defined as mild (Vmax 2.5-2.9), moderate (Vmax 3-3.9), or severe (Vmax ≥4). MI was defined using the fourth universal definition (type 2 MI were not considered).

**Results:**

The mean age was 76.0 years, 54% of the patients were men, and 18.3% had experienced prior coronary event (PCE). At 5 years, the cumulative incidence of MI (death as competing event) was only 2.5% (n = 72, one-third of ST-segment elevation MI). PCE and angina symptoms were associated with an increased risk, whereas female gender was associated with a decreased risk. By contrast, AS severity was not associated with the risk of MI. Subsequent mortality was high and at 52.8% during follow-up (median 648 days after MI occurrence). Incident MI was a powerful predictor of mortality (HR: 2.00, *P* < 0.001 after adjustment).

**Conclusions:**

In patients with AS, the risk of MI is relatively low especially in patients without PCE and without angina. No association between the risk of MI and AS severity was observed. Although rare, incident MI is strongly associated with subsequent mortality.

In the literature, a close interaction between aortic stenosis (AS), atherosclerosis, and coronary artery disease (CAD) has been suggested. Indeed, it has been shown that atherosclerosis and AS share common pathophysiological pathways.^1^^,^^2^ Besides this aspect, these diseases also share similar risk factors; especially atherosclerosis, CAD, and AS are all strongly associated with older age.^1^^,^^3^ In consequence, the prevalence of significant CAD in patients targeted for aortic valve replacement (AVR) was high and up to two-thirds of the patients in main studies and registries,^4^, ^5^, ^6^ although this prevalence decreased in more recent trials focusing on lower risk and younger population.^7^^,^^8^ In addition, concomitant CAD is associated with worse outcomes in patients with severe AS.^9^, ^10^, ^11^ Based on this, preprocedural coronary angiogram (to target patients eligible for subsequent coronary revascularization) is recommended in patients undergoing surgical AVR since concomitant coronary artery bypass graft surgery (CABG) cannot be postponed. However, strategies of systematic coronary revascularization by percutaneous coronary intervention (PCI) in patients undergoing transcatheter AVR differ between centers and are more debated (although still widely used even after the recent publication of the ACTIVATION trial that suggests no benefit of such a strategy).^12^

Nevertheless, if assessing concomitant CAD may be important in such a population, assessing the risk of myocardial infarction (MI) looks even more critical. In this context of a high rate of systematic coronary revascularization before AVR, the risk of periprocedural and postprocedural MI has been reported in several studies and was about 0.5% for the in-hospital course and between 3% and 10% at 5 years after AVR.^13^, ^14^, ^15^, ^16^, ^17^, ^18^ However, the risk of MI in the overall spectrum of AS severity and outside the context of AVR, as well as the correlates and the consequences of the occurrence of MI on outcomes in such a population, has never been extensively studied. In addition, if long-term mortality in AS patients is well described in real-life registries, only few data are available on the long-term incidence of MI.

The VALVENOR multicenter cohort^19^^,^^20^ included 2,830 outpatients with different degrees of AS severity, for whom a 5-year follow-up is available, and therefore presents a perfect opportunity to assess the incidence, correlates, and impact on outcomes of MI occurrence in this specific population in a modern and real-life practice of AS management.

## Methods

### Population

**Figure 1:**
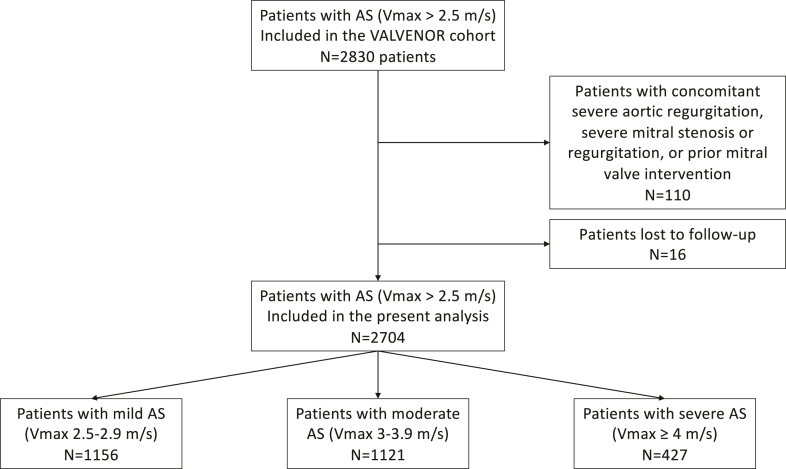
Flow Chart of the Present Analysis AS = aortic stenosis.

As previously published, the VALVENOR study is a multicenter registry that enrolled 2,830 outpatients with native valvular AS between May 2016 and December 2017.^19^^,^^20^ Patients with a peak aortic jet velocity (Vmax) ≥2.5 m/s on transthoracic echocardiography (TTE) were prospectively included by 117 cardiologists from the Nord-Pas-de-Calais region in France during outpatient visits. AS was defined as mild (Vmax 2.5-2.9 m/s), moderate (Vmax 3-3.9 m/s), or severe (Vmax ≥4 m/s).^21^ Patients younger than 18 years or with a documented history of AVR were excluded. We also excluded 110 patients with concomitant severe aortic regurgitation, severe mitral stenosis or regurgitation, or prior mitral valve intervention, leaving 2,720 patients for the present analysis (Figure 1). Participating physicians were selected based on their geographic distribution to provide a representative sample of the current practice of all cardiology care in the region, including university public hospitals, nonuniversity public hospitals, and private practices.

This study was approved by the French medical data protection committee (CCTIRS N°15.526) and authorized by the Commission Nationale de l'Informatique et des Libertés for the treatment of personal health data. All patients consented to the study after being informed in writing of the study's objectives and treatment of the data, as well as on their rights to object, of access, and of rectification.

### Transthoracic echocardiography

TTE was performed as part of routine clinical practice using commercially available systems. Vmax was derived from transaortic flow, recorded with continuous wave Doppler.

### Follow-up

Patients were followed up by their treating cardiologists. The number of outpatient visits was at the discretion of the cardiologists during follow-up. Protocol prespecified follow-up was performed at 2 years and 5 years using a standardized case record form to report clinical events and cardiovascular procedures. To minimize follow-up bias, general practitioners and/or patients were contacted by a research technician in the case of missing information. A 5-year follow-up was completed in 2,704 patients (99.4%). During follow-up, the number of TTEs was left at the discretion of cardiologists.

Incident MI, coronary revascularization, and the cause of death were adjudicated by 2 investigators, with a third opinion in cases of disagreement. For hospitalizations during the follow-up period, hospital records were reviewed for evidence of clinical events. The events reported by the patients were systematically confirmed from the medical reports.

MI was defined according to the fourth universal definition^22^ and categorized as either ST-segment elevation myocardial infarction (STEMI) or non-ST-segment elevation myocardial infarction (NSTEMI). Type 2 MI was not considered as an endpoint for the present analysis. Procedural MI included MI occurring within 48 hours of an index cardiac procedure (coronary angiography, PCI, CABG, surgical AVR, or transcatheter AVR).

Cardiovascular causes of death included complication of AVR, congestive heart failure (HF), sudden death, stroke, limb ischemia, MI, and other means of cardiovascular death. Noncardiovascular causes of death included cancer, sepsis, renal failure, respiratory failure, suicide or accident, and other noncardiovascular death. Deaths by an unknown cause were kept as a separate category.

### Statistical analysis

Continuous variables are described as the mean ± SD except for delays, which are described as median (IQR). Categorical variables are presented as absolute numbers and percentages.

The incidence of MI was estimated with the cumulative incidence function, with death as the competing event.

Univariate and multivariate assessments of variables associated with incident MI were performed with the use of a cause-specific hazard model.^23^ HRs and 95% CIs were calculated. The proportional hazards assumption was tested by visual examination of plots of –ln [-ln (survival time)] against the ln (time) and by including interaction time-dependent terms in the regression analysis.

The association between incident MI and subsequent all-cause mortality was assessed by using a Cox model. In this analysis, incident MI during follow-up was modeled as a time-dependent covariate by the method of episode splitting using the stsplit command in Stata software.

All statistical analyses were performed with Stata version 14.2 (Stata Corp).

## Results

### Study population

As shown in Table 1, this was an elderly population (mean age 76.0 ± 10.8 years), and 54% of the patients were men. A total of 233 patients (8.6%) were recruited from a public university hospital, 760 (28.1%) in nonuniversity public hospitals, and 1,711 (63.3%) by cardiologists in private practice. At inclusion, 1,156 patients (42.7%) had mild AS, 1,121 (41.5%) had moderate AS, and 427 (15.8%) had severe AS.

**Table 1:** Baseline Characteristics of the Study Population (n = 2,704 Patients)

	All Patients (N = 2,704)	No MI (n = 2,632)	MI (n = 72)	*P* Value
Age (y)	76 ± 10.8	76 ± 10.9	77.6 ± 8.9	**0.045**
Women	1,257 (46.5)	1,234 (46.9)	23 (31.9)	**0.014**
Recruitment				
Public university hospital	233 (8.6)	227 (8.6)	6 (8.3)	
Public nonuniversity hospital	760 (28.1)	735 (27.9)	25 (34.7)	0.377
Private practice	1,711 (63.3)	1,670 (63.5)	41 (57)	
History of hypertension	2,066 (76.4)	2,014 (76.5)	52 (72.2)	0.458
Diabetes mellitus	820 (30.3)	793 (30.1)	27 (37.5)	0.142
Prior coronary event	494 (18.3)	462 (17.6)	32 (44.4)	**<0.001**
Prior MI	252 (9.3)	236 (9)	16 (22.2)	**<0.001**
Prior CABG	126 (4.7)	121 (4.6)	5 (6.9)	0.252
Prior PCI	351 (13)	323 (12.3)	28 (38.9)	**<0.001**
Atrial fibrillation	604 (22.3)	581 (22.1)	23 (31.9)	**0.010**
Prior hospitalization for HF	269 (10)	257 (9.8)	12 (16.7)	0.006
Prior stroke	230 (8.5)	226 (8.6)	4 (5.6)	0.477
NYHA fucntional class at inclusion^a^				
I	1,061 (39.5)	1,034 (39.6)	27 (38.6)	
II	1,292 (48.2)	1,255 (48)	37 (52.9)	0.644
III-IV	330 (12.3)	324 (12.4)	6 (8.6)	
Angina at inclusion	109 (4)	99 (3.8)	10 (13.9)	**<0.001**
Peak aortic jet velocity (m/s)	3.27 ± 0.65	3.28 ± 0.66	3.22 ± 0.56	0.523
AS severity at inclusion				
Mild	1,156 (42.7)	1,127 (42.8)	29 (40.3)	
Moderate	1,121 (41.5)	1,084 (41.2)	37 (51.4)	0.098
Severe	427 (15.8)	421 (16)	6 (8.3)	
LVEF at inclusion^b^ (%)	63.8 ± 8.9	63.8 ± 8.8	63.2 ± 9.2	0.267
Beta-blocker	1,210 (44.8)	1,164 (44.2)	46 (63.9)	**0.001**
ACEI or ARB	1,779 (65.8)	1,733 (65.8)	46 (63.9)	0.721
Statin	1,493 (55.2)	1,442 (54.8)	51 (70.8)	**0.012**
Antiplatelet	1,221 (45.2)	1,169 (44.4)	52 (72.2)	**<0.001**
Oral anticoagulant	580 (21.5)	565 (21.5)	15 (20.8)	0.760
Antiplatelet or anticoagulant	1,741 (64.4)	1,678 (63.8)	63 (87.5)	**<0.001**

Values are mean ± SD or n (%). Significant *P* values are highlighted in **bold**.

ACEI = angiotensin-converting enzyme inhibitor; ARB = angiotensin receptor blockers; AS = aortic stenosis; CABG = coronary artery bypass graft surgery; HF = heart failure; LVEF = left ventricular ejection fraction; MI = myocardial infarction; PCI = percutaneous coronary intervention.

a Missing data in 21 patients.

b Missing data in 2 patients.

There was a relatively high prevalence of risk factors and underlying cardiovascular diseases. Altogether, one-third (n = 820 [30.3%]) of the patients had diabetes, and one-fifth (n = 494 [18.3%]) had experienced ≥1 prior coronary event (PCE) (prior MI, n = 252; prior CABG, n = 126; prior PCI, n = 351).

Overall, the cohort was prescribed a broad range of preventive medications with angiotensin-converting enzyme inhibitors or angiotensin receptor blockers in 65.8%, statins in 55.2%, and antiplatelets/anticoagulants in 64.4%.

### Follow-up

Clinical follow-up was completed in 2,704 out of the 2,720 patients (99.4%) at a median of 5.0 [IQR: 3.4-5.5] years.

A total of 1,098 (40.6%) deaths occurred (cardiovascular, n = 452; noncardiovascular, n = 500; unknown cause, n = 146). In detail, there were 435 (37.6%) deaths in patients with mild AS, 467 (41.7%) in patients with moderate AS, and 196 (45.9%) in those with severe AS.

During follow-up, 993 (36.7%) patients underwent an AVR procedure (SAVR, n = 488; TAVR, n = 505). An AVR procedure was performed in 186 (16.1%), 533 (47.5%), and 274 (64.2%) patients with mild, moderate, and severe AS at baseline, respectively.^24^

Coronary angiography was routinely performed as a part of the pre-AVR assessment (480 [98.4%] of the surgical-AVR-treated patients and 496 [98.2%] of the transcatheter-AVR-treated patients had pre-AVR coronary angiography). Altogether, 331 (12.2%) patients underwent coronary revascularization during the overall follow-up period. In total, 373 coronary revascularization procedures were performed in the 331 patients during the follow-up (CABG, n = 127; PCI, n = 246). In most cases (n = 239; 64.1%), coronary revascularization was associated with an AVR procedure. A coronary revascularization was performed in 103 (8.9%), 168 (15%), and 60 (14.1%) patients with mild, moderate, and severe AS at baseline, respectively.

### Risk of MI and correlates

**Figure 2:**
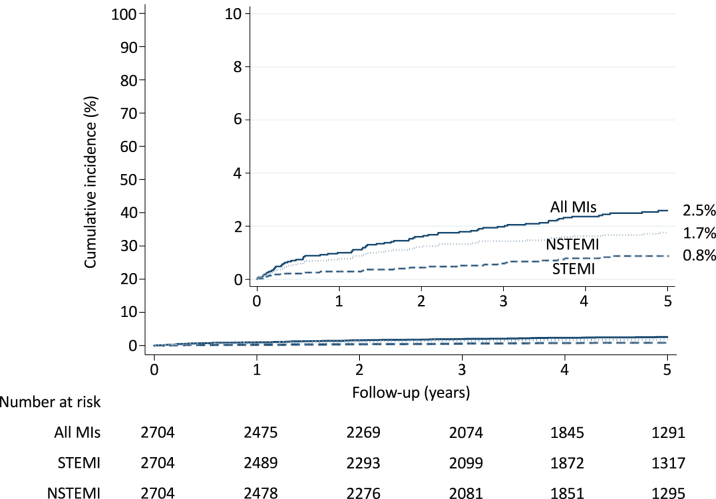
Risk of MI in 2,704 Patients With AS Cumulative incidence of all MI, STEMI, and NSTEMI during the 5-year follow-up (all-cause death as the competing event). MI = myocardial infarction; NSTEMI = non-ST-segment elevation myocardial infarction; STEMI = ST-segment elevation myocardial infarction.

Of the 2,704 patients, 72 presented an MI during the follow-up period. Figure 2 illustrates the 5-year cumulative incidence of MI, with death as the competing event. At 5 years, the cumulative incidence of all MI was 2.5% [95% CI: 2%-3.2%]. Twenty-five MI (34.7%) were categorized as STEMI, and the remainder as NSTEMI. Sixty MI (83.3%) were categorized as type 1 MI while 12 were periprocedural MI (surgical AVR, n = 5; transcatheter AVR, n = 2; CABG, n = 1; PCI, n = 3; coronary angiography, n = 1). Of the 60 patients with type 1 MI, most (n = 56) occurred in patients without AVR while 4 occurred in patients after AVR (2.1, 2.6, 3.9, and 5.3 years after AVR).

**Figure 3:**
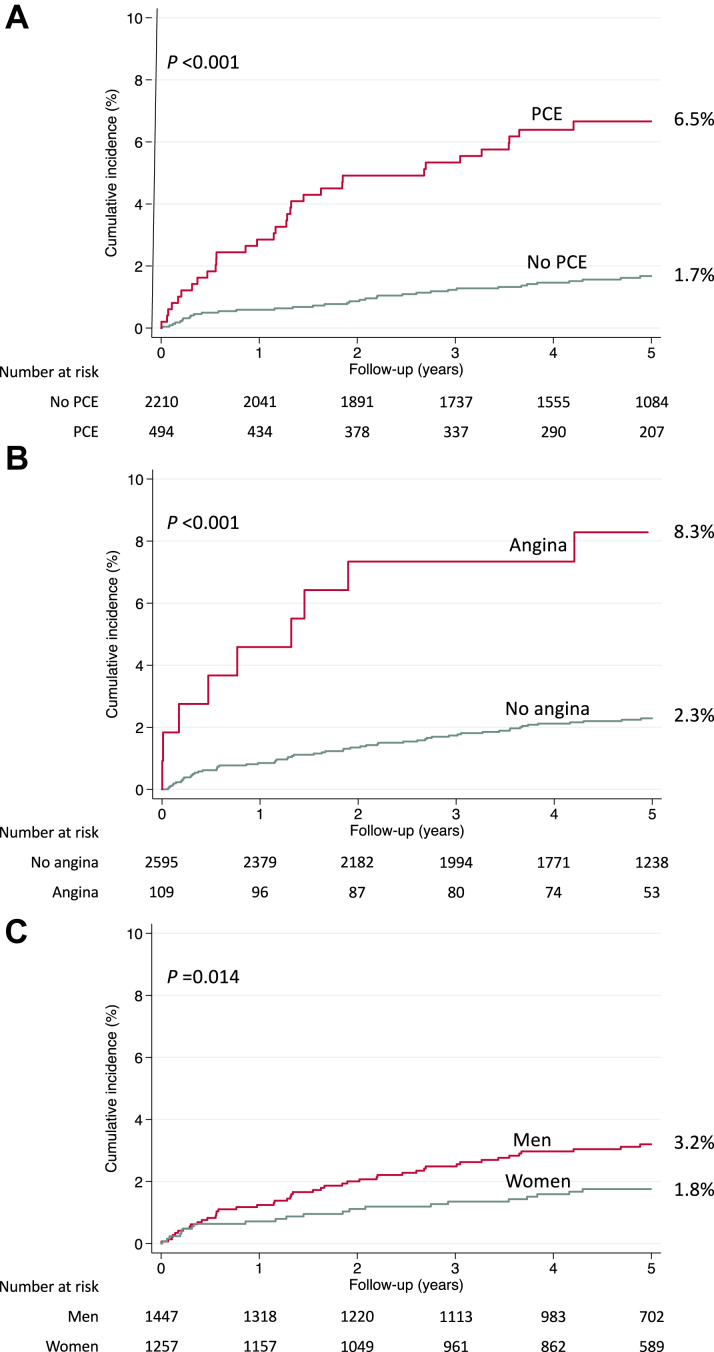
Correlates of MI in Patients With AS Cumulative incidence of MI during the 5-year follow-up according to (A) prior coronary event; (B) angina symptoms at inclusion; (C) gender. PCE = prior coronary event.

**Figure 4:**
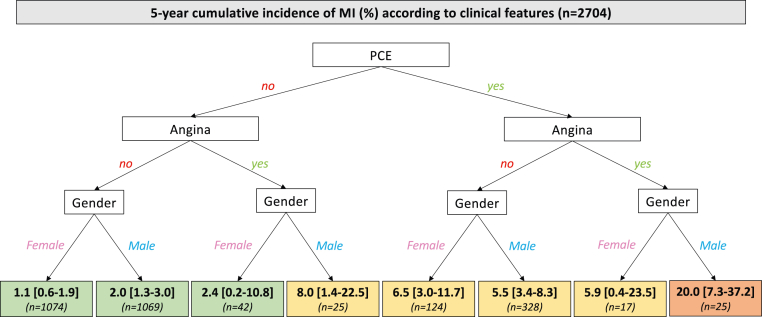
Risk of MI in Patients Stratified by Absence/Presence of a Prior Coronary Event, Absence/Presence of Angina at Inclusion, and Gender MI = myocardial infarction; PCE = prior coronary event.

Univariate and multivariate assessments of baseline variables associated with incident MI are shown in Tables 1 and 2. By multivariate analysis, PCE (*P* < 0.001) and the presence of angina symptoms at inclusion (*P* = 0.001) were associated with an increased risk, whereas female gender was associated with a decreased risk (*P* = 0.041). By contrast, AS severity at inclusion was not associated with the risk of MI. There were 29 (2.5%), 37 (3.3%), and 6 (1.4%) MI cases among patients with mild, moderate, and severe AS at baseline, respectively (Table 1). Figure 3 illustrates the risk of MI according to the 3 independent correlates. Five-year cumulative incidence of MI was 6.5% [95% CI: 4.5%-8.9%] in patients with PCE vs 1.7% [95% CI: 1.2%-2.3%] in patients with no PCE (*P* < 0.001), 8.3% [95% CI: 4.1%-14.4%] in patients with angina vs 2.3% [95% CI: 1.8%-2.9%] in patients without angina (*P* < 0.001), and 3.2% [95% CI: 2.4%-4.2%] in men vs 1.8% [95% CI: 1.1%-2.6%] in women (*P* = 0.014). Figure 4 shows the 5-year risk of MI in patients stratified by presence/absence of PCE, presence/absence of angina at inclusion, and gender. The 2 largest groups of patients were women, without PCE and without angina (n = 1,074, 39.7% of the total population), who had a 5-year cumulative incidence of MI of 1.1% [95% CI: 0.6%-1.9%], and men, without PCE and without angina (n = 1,069, 39.5% of the total population), who had a 5-year cumulative incidence of MI of 2.0% [95% CI: 1.3%-3.0%].

**Table 2:** Independent Predictors of MI Occurrence, Multivariate Analysis

	HR (95% CI)	*P* Value
Prior coronary event	2.80 (1.70-4.59)	**<0.001**
Angina at inclusion	3.36 (1.68-6.70)	**0.001**
Women	0.58 (0.35-0.98)	**0.041**
Age (per year)	1.02 (1.00-1.05)	0.072
Atrial fibrillation	1.48 (0.86-2.55)	0.153
Prior hospitalization for HF	1.48 (0.75-2.92)	0.252
AS severity at inclusion		
Mild	Reference	
Moderate	1.34 (0.82-2.18)	0.244
Severe	0.61 (0.25-1.48)	0.273
Diabetes mellitus	1.29 (0.79-2.11)	0.314

Values are HR (95% CI) by the Cox model. List of variables included in the multivariate model: gender, age, history of prior coronary event, angina at inclusion, history of atrial fibrillation, prior hospitalization for heart failure, aortic stenosis severity at inclusion and history of diabetes mellitus. Significant *P* values are highlighted in **bold**.

AS = aortic stenosis; HF = heart failure.

### MI-associated mortality

**Figure 5:**
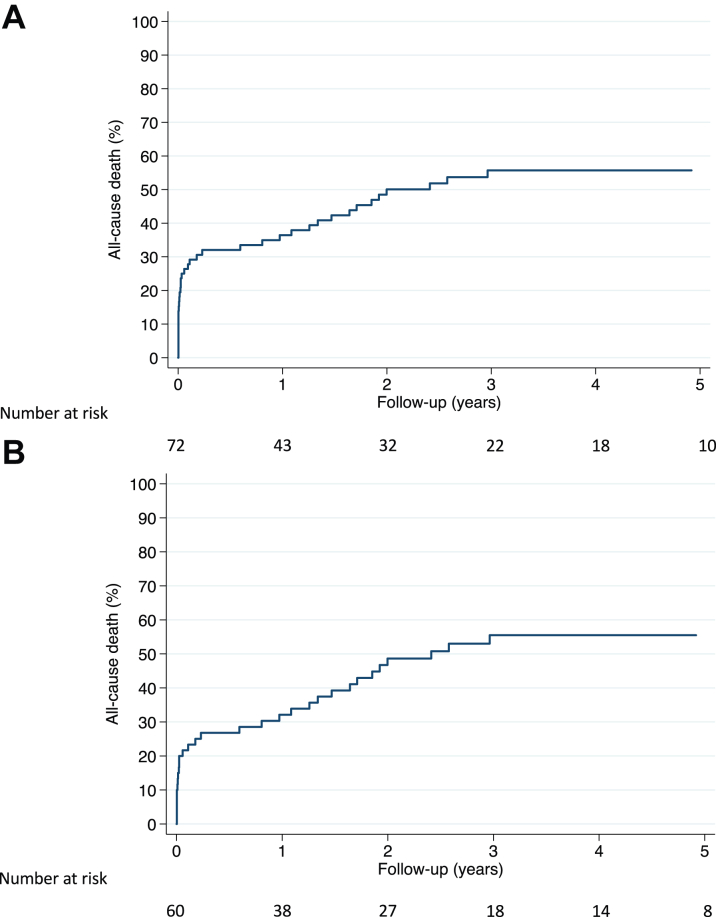
Mortality After MI in Patients With AS (A) Cumulative risk of all-cause death in the 72 patients with incident MI; (B) cumulative risk of all-cause death in the 60 patients with incident type 1 MI (after exclusion of the 12 patients with peri-procedural MI). The follow-up started on the day of MI.t

Of the 72 patients with incident MI, 38 (52.8%) died during follow-up (median 648 [IQR: 17-1,417] days after MI occurrence). When analyzed as a time-dependent variable, incident MI was a powerful predictor of all-cause mortality (unadjusted HR: 2.56 [95% CI: 1.85-3.55], *P* < 0.001). Incident MI remained significantly associated with mortality (HR: 2.00 [95% CI: 1.44-2.79], *P* < 0.001) after adjusting for age, gender, diabetes, PCE, atrial fibrillation, prior hospitalization for HF, prior stroke, angina at inclusion, AS severity, and left ventricle ejection fraction. Figure 5A shows the cumulative risk of mortality after MI in the 72 patients. The mortality rate at 1 year after MI was 36.9% [95% CI: 26.8%-49.2%]. Figure 5B shows same data in the 60 patients with type 1 MI (after exclusion of the 12 patients with peri-procedural MI).

## Discussion

The originality of the present study is that it focuses on 2,704 outpatients with a large spectrum of AS severity (and not solely on patients with severe AS) who were included in a prospective region-wide and modern registry and with a long-term follow-up of 5 years. The main results of our study can be summarized as follows (Central Illustration): 1) in the overall study population, the risk of incident MI was relatively low (2.5% at 5 years), and this risk was even lower in patients without PCE and without angina (Central Illustration B and C); 2) most MI (83%) occurred outside the context of AVR procedures, and there was no association between the risk of MI and AS severity (Central Illustration D); and 3) incident MI was strongly associated with subsequent mortality in this subset of patients (Central Illustration B).

**Central Illustration:**
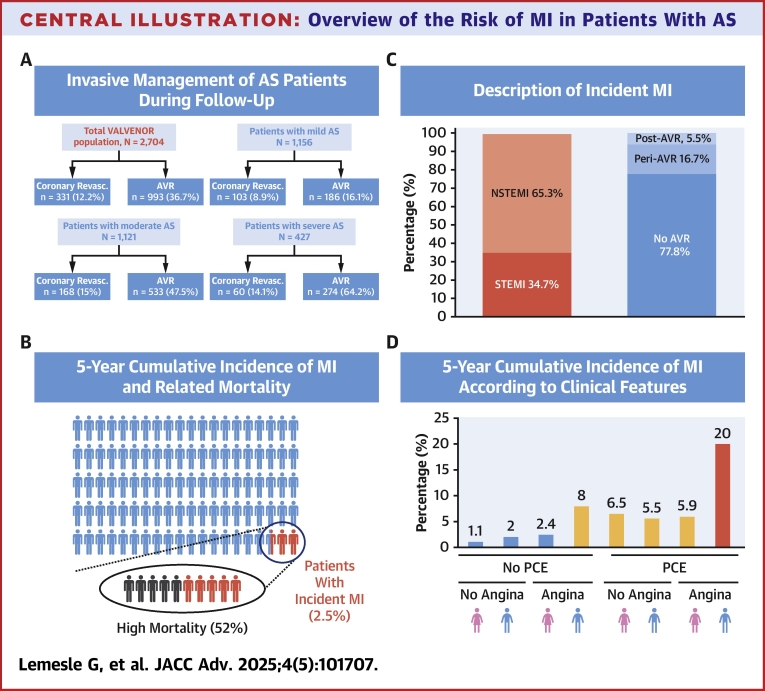
Overview of the Risk of MI in Patients With AS (A) invasive management of as patients during follow-up; (B) 5-year cumulative risk of MI and related mortality; (C) description of MI events; (D) 5-year cumulative risk of MI stratified by absence/presence of prior coronary event, absence/presence of angina at inclusion, and gender. The follow-up started on the day of MI. AS = aortic stenosis; AVR = aortic valve replacement; MI = myocardial infarction; NSTEMI = non-ST-segment elevation myocardial infarction; STEMI = ST-segment elevation myocardial infarction; PCE = prior coronary event.

Previous studies have suggested that AS and CAD share common pathophysiological mechanisms.^1^^,^^2^ Furthermore, risk factors for AS, such as older age, male sex, history of hypertension, smoking, and low-density lipoprotein cholesterol, have been shown to be similar to those for atherosclerosis and therefore CAD.^1^^,^^3^ Subsequently, it has for long been demonstrated that significant CAD is often present in patients with severe AS at the time of AVR^4^, ^5^, ^6^ and more importantly that the presence of concomitant significant CAD negatively impacts prognosis in these patients after AVR.^9^, ^10^, ^11^ By contrast, the true prevalence of significant CAD in patients with non-severe AS (ie, mild or moderate) is poorly described. In our study, PCE (which may probably underestimate the presence of asymptomatic significant CAD) was present in only 18.3% of the patients in the overall population, which is much lower than what was observed in patients with severe AS targeted for AVR in the literature.^4^^,^^5^

In this context, it looks therefore critical for physicians to specifically assess the risk of MI in this subset of patients. To date, this risk has been largely studied in the peri-AVR and post-AVR periods and up to 5 years.^13^, ^14^, ^15^, ^16^, ^17^, ^18^ By contrast, data on the actual risk of MI in AS patients at distance of any AVR procedure (ie, excluding periprocedural events) are lacking. Although the frequent association of CAD with AS may theoretically argue for a high risk of acute coronary events in this population, this has never been studied prospectively.

Based on our results, it should first be noted that MI related to AVR procedures are relatively infrequent (7 MI for 993 AVR, ie, 0.7%). This result is in agreement with previous investigations of patients undergoing AVR in whom the risk of periprocedural MI was in the 0.5% to 1% range.^13^^,^^17^ Importantly, these data should be interpreted in a context of systematic pre-AVR coronary angiography followed by revascularization when indicated by local heart teams, as recommended by guidelines.^25^, ^26^, ^27^ In our population, this strategy was predominant although the place of systematic coronary angiography and subsequent revascularization is to date debated when patients are targeted for transcatheter AVR.^5^^,^^12^ Indeed, transcatheter AVR offers the advantage of being able to defer the treatment of concomitant CAD when clinically relevant.

In facts, the vast majority of MI were not related to AVR procedures and were indeed distributed throughout the follow-up period. Of main importance, there was no evidence of a relationship between AS severity and the risk of MI. To the best of our knowledge, the present study is the first prospective assessment of incident MI in this particular population, and our observation of a 2.5% cumulative incidence of MI after 5 years will be a benchmark for future studies. This relatively low risk of MI despite pathophysiological evidence linking AS and the development of CAD may, at least in part, be related to regular clinical follow-up because of AS diagnosis and to the high rate of prescription of preventive cardiovascular medications. In our study, two-thirds of the patients were indeed prescribed at least one antithrombotic at inclusion and 55% a statin therapy. As a point of comparison, the cumulative incidence of MI was 4% at 5 years in the CORONOR registry (mean age 67 ± 12 years) that included stable outpatients with chronic CAD from the same region in France.^28^ Of note, we identified 3 baseline characteristics (PCE, angina symptoms, and male gender) as independent correlates of MI. Again, AS severity was not associated with the risk of MI. Risk stratification, which is an integral part of management for all types of cardiovascular diseases, may thus help to predict incident MI in AS patients. Although the combination of risk factors selected high-risk patients, our data also highlighted that most patients (80%) belong to low-risk categories with 5-year cumulative incidence of MI of 1.1% in women and 2% in men in the absence of PCE and angina symptoms. Our data therefore suggest that the risk of MI in AS patients with no PCE and no angina, despite their relatively old age, is particularly low (around 0.3% per year). Noninvasive systematic detection of underlying CAD in these patients outside the context of AVR may therefore seem of little relevance in daily practice.

Finally, although a relatively rare event, incident MI in AS patients was associated with high mortality rate (one-third of the patients died within 1 year). This was still observed when procedural events (mostly in a context of AVR) were excluded. This rate was particularly high and much higher than what was observed in stable CAD patients included in the CORONOR registry who experienced a mortality rate of 20% after a median follow-up of 821 days following the intercurrent MI.^28^ The exact reasons underlying this poor outcome are unknown, but several hypotheses might be proposed. First, it may be speculated that the risk of MI complications is increased when occurring in patients with AS, especially the risk of HF. Second, comorbidities (age, risk factors, associated cardiovascular diseases) might have played a role. Third, AS has been shown to be associated with chronic myocardial ischemia, left ventricle hypertrophy, and subsequent coronary flow impairment.^29^, ^30^, ^31^ In a recent study including 1,373 patients with MI, moderate AS was associated with higher risk of HF and mortality at 1 year in both STEMI and NSTEMI patients.^31^ Singh et al^29^ have shown similar results with a higher risk of death in patients with concomitant STEMI and AS than in patients with STEMI and no AS.

### Strengths and limitations

The VALVENOR registry was conducted in the region Nord-Pas-de-Calais in France, and our results may not be extrapolated to other countries with higher or lower prevalence of CAD. Although a protocol prespecified follow-up was performed at 2 years and at 5 years using a standardized case record form to report clinical events and cardiovascular procedures, the number of visits and TTEs during follow-up was left at the discretion of the treating cardiologists. Of note, although scheduled coronary revascularization (in patients with chronic coronary syndrome) has not been associated with a strong and clear decrease in the risk of MI in the literature,^32^^,^^33^ it should be emphasized that the relatively high proportion of coronary angiogram and subsequent coronary revascularization performed in patients targeted for AVR during follow-up may have influenced the medical treatment of the patients with severe AS and therefore decrease the risk of MI. Finally, our dataset was not designed to answer the question whether AS increases or not the risk of MI as compared to a population without AS. Despite these limits, the VALVENOR study offers the opportunity to extensively assess the risk of MI in AS patients and in a real-life and modern practice.

## Conclusions

In patients with AS, the risk of MI is relatively low especially in patients without PCE and without angina. Of note, no association between the risk of MI and AS severity was observed in the present study. Based on this, outside the context AVR, systematic detection of underlying CAD looks not relevant in daily practice in asymptomatic patients with AS. Although rare, incident MI is however strongly associated with subsequent mortality.

Perspectives **COMPETENCY IN MEDICAL KNOWLEDGE 1:** In AS patients, the risk of MI is relatively low (about 2.5% at 5 years), and this risk is even lower in AS patients without PCE and without angina.

**COMPETENCY IN MEDICAL KNOWLEDGE 2:** The risk of MI is not associated with the degree of severity.

**COMPETENCY IN MEDICAL KNOWLEDGE 3:** Even rare, the occurrence of MI in patients with AS is associated with a poor prognosis.

**COMPETENCY IN PATIENT CARE:** Noninvasive systematic detection of underlying CAD in asymptomatic patients with AS and no PCE outside the context of AVR seems of little relevance in daily practice.

**TRANSLATIONAL OUTLOOK 1:** Further studies are required to assess if AS is associated or not with a higher risk of MI as compared to patients without AS (Vmax <2.5 m/s).

**TRANSLATIONAL OUTLOOK 2:** In the next future, ongoing trials may give the answer whether or not systematic coronary angiogram and subsequent coronary revascularization is beneficial in the context of AVR.

## Funding support and author disclosures

This study was supported by a grant from 10.13039/501100003100Fédération Française de Cardiologie. All authors have reported that they have no relationships relevant to the contents of this paper to disclose.

## References

[1] Abdul-Rahman T., Lizano-Jubert I., Garg N. (2023). The common pathobiology between coronary artery disease and calcific aortic stenosis: evidence and clinical implications. Prog Cardiovasc Dis.

[2] Milin A.C., Vorobiof G., Aksoy O., Ardehali R. (2014). Insights into aortic sclerosis and its relationship with coronary artery disease. J Am Heart Assoc.

[3] Stewart B.F., Siscovick D., Lind B.K. (1997). Clinical factors associated with calcific aortic valve disease. Cardiovascular Health Study. J Am Coll Cardiol.

[4] Faroux L., Guimaraes L., Wintzer-Wehekind J. (2019). Coronary artery disease and transcatheter aortic valve replacement: JACC State-of-the-Art Review. J Am Coll Cardiol.

[5] Tarantini G., Tang G., Nai Fovino L. (2023). Management of coronary artery disease in patients undergoing transcatheter aortic valve implantation. A clinical consensus statement from the European Association of Percutaneous Cardiovascular Interventions in collaboration with the ESC Working Group on Cardiovascular Surgery. EuroIntervention.

[6] Iung B. (2000). Interface between valve disease and ischaemic heart disease. Heart.

[7] Popma J.J., Deeb G.M., Yakubov S.J. (2019). Transcatheter aortic-valve replacement with a self-expanding valve in low-risk patients. N Engl J Med.

[8] Mack M.J., Leon M.B., Thourani V.H. (2019). Transcatheter aortic-valve replacement with a balloon-expandable valve in low-risk patients. N Engl J Med.

[9] Minten L., Wissels P., McCutcheon K. (2022). The effect of coronary lesion complexity and preprocedural revascularization on 5-year outcomes after TAVR. JACC Cardiovasc Interv.

[10] Stefanini G.G., Stortecky S., Cao D. (2014). Coronary artery disease severity and aortic stenosis: clinical outcomes according to SYNTAX score in patients undergoing transcatheter aortic valve implantation. Eur Heart J.

[11] Aurigemma C., Massussi M., Fraccaro C. (2023). Impact of chronic coronary artery disease and revascularization strategy in patients with severe aortic stenosis who underwent transcatheter aortic valve implantation. Am J Cardiol.

[12] Patterson T., Clayton T., Dodd M. (2021). ACTIVATION (PercutAneous coronary inTervention prIor to transcatheter aortic VAlve implantaTION): a randomized clinical trial. JACC Cardiovasc Interv.

[13] Beyersdorf F., Bauer T., Freemantle N. (2021). Five-year outcome in 18 010 patients from the German aortic valve registry. Eur J Cardiothorac Surg.

[14] Van Mieghem N.M., Deeb G.M., Sondergaard L. (2022). Self-expanding transcatheter vs surgical aortic valve replacement in intermediate-risk patients: 5-year outcomes of the SURTAVI randomized clinical trial. JAMA Cardiol.

[15] Gleason T.G., Reardon M.J., Popma J.J. (2018). 5-year outcomes of self-expanding transcatheter versus surgical aortic valve replacement in high-risk patients. J Am Coll Cardiol.

[16] Mack M.J., Leon M.B., Smith C.R. (2015). 5-year outcomes of transcatheter aortic valve replacement or surgical aortic valve replacement for high surgical risk patients with aortic stenosis (PARTNER 1): a randomised controlled trial. Lancet.

[17] Auffret V., Lefevre T., Van Belle E. (2017). Temporal trends in transcatheter aortic valve replacement in France: France 2 to France TAVI. J Am Coll Cardiol.

[18] Makkar R.R., Thourani V.H., Mack M.J. (2020). Five-year outcomes of transcatheter or surgical aortic-valve replacement. N Engl J Med.

[19] Coisne A., Aghezzaf S., Butruille L. (2023). Incidence, source, and prognostic impact of major bleeding across the spectrum of aortic stenosis. Am Heart J.

[20] Coisne A., Montaigne D., Aghezzaf S. (2021). Association of mortality with aortic stenosis severity in outpatients: results from the VALVENOR study. JAMA Cardiol.

[21] Baumgartner H., Falk V., Bax J.J. (2017). 2017 ESC/EACTS Guidelines for the management of valvular heart disease. Eur Heart J.

[22] Thygesen K., Alpert J.S., Jaffe A.S. (2018). Fourth universal definition of myocardial infarction (2018). Circulation.

[23] Wolbers M., Koller M.T., Stel V.S. (2014). Competing risks analyses: objectives and approaches. Eur Heart J.

[24] Coisne A., Montaigne D., Aghezzaf S. (2024). Clinical outcomes according to aortic stenosis management: insights from real-world practice. J Am Heart Assoc.

[25] Writing Committee M., Lawton J.S., Tamis-Holland J.E. (2022). 2021 ACC/AHA/SCAI guideline for coronary artery revascularization: a report of the American College of Cardiology/American Heart Association joint committee on clinical practice guidelines. J Am Coll Cardiol.

[26] Vahanian A., Beyersdorf F., Praz F. (2022). 2021 ESC/EACTS guidelines for the management of valvular heart disease. Eur Heart J.

[27] Writing Committee M., Otto C.M., Nishimura R.A. (2021). 2020 ACC/AHA guideline for the management of patients with valvular heart disease: a report of the American College of Cardiology/American Heart Association joint committee on clinical practice guidelines. J Am Coll Cardiol.

[28] Lemesle G., Tricot O., Meurice T. (2017). Incident myocardial infarction and very late stent thrombosis in outpatients with stable coronary artery disease. J Am Coll Cardiol.

[29] Singh G.K., van der Bijl P., Goedemans L. (2021). Prevalence of aortic valve stenosis in patients with ST-segment elevation myocardial infarction and effect on long-term outcome. Am J Cardiol.

[30] Patlolla S.H., Maqsood M.H., Belford P.M. (2022). Impact of concomitant aortic stenosis on the management and outcomes of acute myocardial infarction hospitalizations in the United States. Am Heart J Plus.

[31] Abraham B., Farina J.M., Fath A. (2023). The impact of moderate aortic stenosis in acute myocardial infarction: a multicenter retrospective study. Catheter Cardiovasc Interv.

[32] Maron D.J., Hochman J.S., Reynolds H.R. (2020). Initial invasive or conservative strategy for stable coronary disease. N Engl J Med.

[33] Boden W.E., O'Rourke R.A., Teo K.K. (2007). Optimal medical therapy with or without PCI for stable coronary disease. N Engl J Med.

